# A Clinical Report of Cancer Cases Treated with Radium

**Published:** 1915-07

**Authors:** John M. Lee

**Affiliations:** Rochester, N. Y.


### A Clinical Report of Cancer Cases Treated With Radium.

By JOHN M. LEE, M. D., Rochester, N. Y.

This report includes by no means all the cases treated at the Lee Hospital. Most of the incurable ones that have come under our observation have already been recorded elsewhere. When we took these hopeless patients they were informed that in all probability we would be unable to do more than to prolong their lives and relieve their excruciating pain, hemorrhages or other serious symptoms. We have been amazed at the effects of the agent on hitherto hopeless patients: almost indescribable sloughing surfaces of soft parts, cartilages of the nose and ears and bones of the face, have been healed, and clinical cures secured so far as possible with such extensive destruction of tissue.

Case 1. Recurrent cancer of the breast which involved the tissues over an area of five inches around and about the scar just below the border of the axilla. Two applications of radium in three tubes and a varnish applicator equa-distant over the surface, five weeks apart, completely despatched the disease, and the surface is left in a soft, healthy condition.

Case 2. Recurrence after vaginal hysterectomy. The glands were enlarged in the vault of the vagina and grew until they averaged about half an inch in diameter. Radium was applied by tubes very carefully screened. The disease subsided so that no sign of it could be discovered on careful examination. She was regarded as clinically cured at the end of the second month.

Case 3. Epithelioma of the lip first noticed in September, 1914. Treatment afforded no relief and he was advised to enter this hospital. The disease responded nicely to radium and at the end of four weeks he was discharged from the hospital clinically cured. This is the only operable case in which radium has been used, and he refused to be treated by the knife.

Case 4. Recurrent carcinoma of the breast. She had had four operations in quick succession since the breast was removed. The disease returned three months following the last operation, radium was employed and it disappeared after treatment of two months. It is now two and one-half years since the malady was declared to be clinically cured, and she is as sound today as she was when she returned home after the treatment.

Case 5. Developed lupus vulgaris of the face a year ago last spring. A physician advised immediate removal but as he believed it was not serious it was allowed to run on until last April, when he entered this hospital. The use of a halfstrength varnish applicator promptly despatched the growth in part but it was not until seventy-five miligrams of pure radium element were employed that the disease was brought under control and the patient clinically cured.

Case 6. Several years ago noticed a small tumor under the arm and another in the breast. She allowed the disease to run on until it was well developed, when she consulted a prominent surgeon who removed the breast with the pectoral muscles and objectional tissues of the axillary space. The disease returned and she applied for radium treatment last February. About two hundred milligrams of radium salts promptly despatched the disease and she was clinically cured within a period of two months.

Case 7. Developed a small aptheous ulcer on his tongue five years ago. The Wasserman test was negative and treatment was unsuccessful. The histological examination showed the disease to be epithelioma. X-Ray treatment was employed for two years, nevertheless the disease kept spreading until the entire organ was involved. He entered this hospital and tratment was immediately begun by stitching radium tubes on the tongue. These were employed at intervals of from four to six weeks and within eight months the disease appeared to be eradicated.

Case 8. Squamous cell epithelioma between the lower jaw and face, appeared to be due to pressure from the lower plate of his false teeth. The cross-fire method relieved the excruciating pain and the tumor immediately decreased in size. The disease disappeared and he remaind clinically cured for a number of weeks when it reappeared. He is now under treatment for the second time and we shall be able to arrest the disease but what the final result will be cannot be foretold as the squamous cell variety of cancer is exceedingly difficult to treat.

Case 9. Rodent ulcer of the face and nose. It gradually increased in size, even under the care of a number of physicians. When I received her, July 15, 1913, a well developed rodent ulcer of three year's duration occupied the lower third of the left side of the nose and extended to the cheek as far as the external canthus. A twelve hour application of a one-half strength varnish applicator screened by a piece of brass one millimeter and rubber two millimeters in thickness, respectively, completely arrested the disease and she remains well at the present time.

Case 10. About a year ago noticed an irritation of the upper gum, apparently from her false teeth the plate of which was cracked. She says she foolishly kept on wearing it until the last of May, 1914, when she consulted her son who is a physician. He advised her to take out the plate, but was unable to heal the ulcer and it finally developed into epithelioma commonly known as epulus. It was removed by operation but it immediately returned. She was sent to this hospital and treatment was begun October 1, 1914, and she was discharged clinically cured February 28, 1915.

Case 11. Noticed a bunch in his lip which he believed was caused by smoking a pipe. The disease was removed by the plaster method about two years ago but it did not heal: the mass gradually enlarged until it was about the size of a hen's egg. For a time it was very hard, but a few weeks before he consulted me it broke down and became painful. There were two ways to treat it: by removing the under lip and constructing a new one; and the use of radium. He chose the latter, and radium was applied December 7th and he was discharged clinically cured March 3, 1915.

Case 12—Noticed a little bunch on her jaw five years ago but paid no attention to it until some time after when she consulted a surgeon who removed the "glands of her neck" and pronounced the disease to be spindle cell sarcoma. There was not much trouble for a year or two when the disease returned and a surgeon removed the growths in part, but as they involved the tonsil, side of the cheek, sub-lingual glands and the gums, he was obliged to abandon the operation. As the months passed her sarcomata developed markedly in size and when she entered this hospital radium was employed externally and internally and the disease caused to disappear.

Case 13. Had extensive epithelioma of the tongue. He had employed X-Ray treatment among other remdies for two years without improvement. When he entered this hospital the entire tongue was involved with a whitish nodular mass and his case had been pronounced hopeless by a number of physicians. Histological examination showed it to be squamous cell epithelioma. The treatment extended over a period of six months; a number of times three tubes and a varnish applicator carrying nearly two hundred milligrams of radium salts were stitched on his tongue and allowed to remain twenty-four hours. The disease was exceedingly obstinate, as is the rule with this variety of cancer; this condition in a very sensitive patient made his treatment difficult and tedious, though a good result was obtained.

Case 14. Recurrent carcinoma of the breast. It was removed eighteen months prior to her entrance to this hospital. The disease had developed throughout the line of the scar and the enlarged glands were intimately united to the axillary vein. The disease tissue was removed by a liberal dissection and the wound closed. After the first dressing was done raduim was applied and kept an inch away from the line of union and applied for four hours in a place until three tubes and a varnish applicator a half inch apart were made to cover all the surface in the axilla and for six inches each side of the scar. The healing process just below the axilla was interrupted somewhat though it was not at all troublesome, and after a time closed.

Case 15. Noticed an enlargement in her breast about the last of October, 1913, and consulted a surgeon who advised removal of the gland together with pectoral muscles and the clearing of the axillary space, etc., and the operation was thoroughly carried out. The next September the disease returned and she had a second operation in October, 1914. Within a few weeks the medullary form of the disease appeared in the skin and subcutaneous tissues and spread with such rapidity that remedial measures became immediately necessary. A third operation was declined by her surgeon, as he regarded her case hopeless. She entered this hospital December 22, 1914, and radium was applied. It was repeated every three or four weeks for a period of three months. The disease was very obstinate, as is always the case with medullary carcinoma, though marked progress was registered. One morning while her daughter was in the room and they were preparing to return home in their automobile, for several weeks, she was stricken with apoplexy and died that evening.

Case 16. Two years ago noticed a tumor developing on the inside of his lip. The disease progressed until the lip was perforated rather low on the chin where a tumor developed the size of a walnut. He dispensed with his false plate, which he believed caused sufficient irritation to develop the disease. The condition became alarming and he consulted his family physician, who took him to one of the leading hospitals and operation was refused because of the wide dissemination of the disease on both sides of the jaw and overlying tissues. The surgeon and the family physician brought him to this hospital and radium was immediately employed. The disease responded promptly and the progress has been steady and satisfactory. The opening through the chin and the excrescence have disappeared and all the disease inside of the mouth so far as is possible by the use of this agent has healed. The bone which was partially destroyed remains bare and will probably have to be chiseled off sufficiently to allow the tissues to cover. Aside from this he is in excellent condition.

Case 17. Lupus of the abdomen, cancer of the foot, enlarged, cancerous inguinal glands, passive congestion and very marked edema of the leg. Although the case was most forbidding, after the facts were presented to the patient she desired radium treatment. She was never very well as a girl or young woman and from eighteen to thirty-five she had been an invalid most of the time. When twenty, the cervical glands became enlarged and suppurated and large scars remained at the side of the neck. About six years ago lupus vulgaris of the abdomen developed and shortly thereafter it appeared on the foot near the inner side of the great toe. She received good treatment of various kinds, including the X-ray, for many months and was not benefitted. Finally a surgeon removed the first and second toes and believed she would not have serious trouble thereafter. A year later, in October, 1914, a surgeon advised her to have the right foot amputated. This was declined. A little later another competent operator proposed amputation at the hip joint. This was also declined and she entered this hospital. Wasserman examination was negative and it was clear that the lupus of the foot had undergone carcinomatous degeneration and the whole appearance of her case was most forbidding; still applications of the element to the enlarged glands over the femoral veins caused such marked shrinkage as to permit the circulation to be nearly normal and the edema was greatly lessened. The lupus of the abdomen disappeared and the hard ring of indurated tissue about the ulcer of the foot, characteristic of X-ray treatment, gradually yielded and reparative action was taking place when she developed embolism and died suddenly.

Case 18. Three years ago developed cancer over the sternum between the mammae, also over the jugular veins of the right side. She was a patient in two of the local hospitals for a number of months and operation was, without the use of radium, wisely refused. Finally X-ray treatments were employed during one whole summer. When she entered this hospital an open ulcer occupied all the space between the mammae and the clavicle and down to a point half way to the ensiform cartilage. The center of this ulcer had healed over a surface one inch in diameter and all around this it was open; the skin and underlying tissues were elevated and in shape and size resembled a quoit and were almost of a stony hardness This ring of cancerous tissue and the ulcer were removed even from between the ribs as far as possible and a large flap turned from the left breast into the open wound and stitched. Two weeks later when this flap had healed, the mass on the neck was extirpated with a section of the jugular veins which were involved in the growth. The clavicle was divided and retracted so as to make room to tie the jugulars near their origin. A twenty-five milligram tube of pure radium element screened with glass, silver and lead tubes was placed into the end of a thick drainage tube and carried down to the bottom of the wound and left for four hours as a prophylactic. When the wound had healed radium was used to destroy any cancerous remnants which might exist. The wound in the region of the radium tube healed tardily and left several sinuses, one of which led to the divided clavicle which exfoliated several pieces of bone; then the sinus healed. Radium was used five times, a month apart, and the wounds have all healed and an excellent result is secured.

Case 19. Cancer of the right tonsil, of the gum and inner surface of the soft tissues over the inferior maxilla. Three tubes over which a good size rubber glove finger was carried and securely fastened around braided silk threads which were tied to the proximal ends of the tubes. Another piece of braided silk was tied to the rubber close to the distil ends of the tubes; a catheter was passed through the right nasal cavity into the pharynx and drawn forward; the braided silk threads attached to the proximal ends of the tubes were secured to the catheter and drawn out through the anterior nares and clamped with a pair of artery forceps: the other threads securely tied to the rubber glove finger at the distil ends of the tubes were threaded in a needle and carried through the tissues of the chin under the jaw just back of its angle. The tubes were then drawn up so as to nicely come over the tonsil and the threads tied over a small roll of rubber adhesive plaster which was drawn up against the nose so as to hold the tubes at the desired position on the tonsil; then the other braided silk threads which had been carried through the side of the chin were drawn up snug so as to bring the tubes over the cancerous ulcer and these were in turn tied down over a small roll of rubber plaster with the adhesive side outward against the skin. The tubes were left in this site twenty hours, then drawn down along the inside of the jaw and allowed to remain twenty more hours. In this way all the surfaces were rayed. During this process cross-fire was established by another set of tubes applied exactly opposite these on the neck and along the outer surface of the jaw. This application caused all the surfaces to heal and the patient was clinically cured.

Since the removal of tissue from cancerous growths tends to disseminate the disease we have refrained from making examinations except in favorable cases; therefore the histological make-up of the above cases is incomplete and often valuable and scientific information is thereby lost.

Rodent ulcer is certainly one of the readily curable forms of cancer, but if allowed to "run on" metastasis is so frequent that the final results are scarcely less to be dreaded than the more malignant varieties of the disease. It was the success with which radium controlled rodent ulcer and epithelioma that encouraged me to purchase radium in rather large quantities and prepare myself to treat any disease in which the element is indicated. Certainly it acts very effectively in all the forms of carcinoma that I know of except two, the squamous cell and the medullary varieties of the disease and one need not always despair of success even in these. It is also of inestimable value in its sister disease sarcoma, especially the round cell variety. In the tongue cases I was unable to secure prompt results except when the tubes were stitched to the organ and allowed to remain from twelve to twenty-six hours. A number of cases which developed in the mouth, about the gums, the tongue and other sites were clearly traceable in many instances to irritation from badly fitting or broken false teeth, jagged teeth as well as to smoking.

Epithelioma or sarcoma of the tonsils and surrounding tissues seems to have yielded more readily to the treatment than the disease in some other sites about the face.

While on one of my trips to another city to investigate the value of radium-therapy, a man of wide experience casually remarked one day, while walking over the wards of a large hospital, that he dreaded to get a case of cancer which had previously been treated by the X-ray. "In fact," he said, "I feel as though I would never take another." I did not comment upon the reasons why but since I have been working with radium they have come to me very forcefully. I have had quite a large number of patients in whom the X-ray treatment had been employed for from a few months to two years and I cannot help but feel that they were much more difficult to bring under the beneficial effects of radium-therapy than others. None of these credit the method with any improvement, and all of them believe that the disease grew gradually worse under its use. In many of the cases there have been elevated lines of from half-an-inch to an inch wide surrounding the growths or ulcers, almost of a stony hardness and in one instance resembled in shape and size a quoit ring around the ulcer, the center of which had cicatrized. Under these hard tissues there is a line of dark semi-gangrenous tissue, even under healed portions, which prevents sound repair and it is not until all this is destroyed that successful results can be secured. In the cases in which this peculiar condition was not present, the X-ray treatments were not of long duration.

We generally have a number of such cases under treatment and when the ring of hardened tissue is considerable it is easier and better to remove it with the knife and then use the radium on such parts of the disease as cannot be extirpated. Any way the cases may be managed, radium treatment is attended by infinitely greater difficulties where the X-ray has been used than in any others that have fallen to our lot to manage; though if large dosage be employed throughout a long period, the disease can finally be arrested and the wounds healed by the use of the element alone. Other bad results often follow the use of the Roentgen ray. One especially sad case comes to my mind at this moment, in which a lady had undergone treatment for the cure of extensive lupus vulgaris of the face. The soft parts were burned throughout the entire side of the face and when healing finally was secured, at the end of fourteen months, the contraction was so great that the under eyelid was completely everted and the mouth drawn to one side. Atrophic changes had taken place to such an extent that the face had a marked one-sided appearance. Islands of lupus still remain in the cicatrix and the disease is disseminated about the scar and on the other side of the face. There are interspersed also enlarged vessels which resemble markedly fairly extensive angioma and the unsightliness of the face baffles description. Not all of these results often follow the use of the X-ray in a single case, but very frequently some of them are present.

We have the latest pattern of X-ray machine in our office but have not used it in the treatment of the various forms of the red plague because we have not regarded the method indicated in these diseases and unless one is equipped with the Coolidge tube, suitable screens, and all modern apparatus, this plan of treatment better be laid aside altogether. Even with this latest technique the machine is not suited to many forms of cancer, therefore it will, if finally found successful, best be used to supplement the use of radium in a restricted number of cases. This new element is much more penetrating than the X-ray and therefore more satisfactory in its results. I know that in this view I am not supported by all, but it is because some surgeons work with too small dosage, as I have already pointed out in previous articles published in the North American Journal and the Medical Century for June 1915. A marked illustration of just what I mean may be found in an article by Joseph C. Beck, M. D., published in the Journal of the Indiana State Medical Association for May 1915, at page 236. This able surgeon says: "In deeper seated malignant disease, particularly carcinoma, my results were absolutely negative as to cure by simple application of radium; but by applying the radium for a week or two daily for one hour over the various locations of the growth and then operating, but not radically, and then again applying the radium post-operative, I believe there is some hope."

This dosage of radium would aggregate one hundred forty to two hundred eighty milligram hours radiation (radium element) during the course of treatment at the maximum and under the above quoted plan might be very much less; from my view point I cannot see how any one can expect any result whatever from this exceedingly meager dosage. In my judgment, "deeper seated malignant disease, particularly carcinoma," requires a very different technique from that employed by Dr. Beck, and his dosage should be multiplied by forty, which would increase the maximum milligram hours radiation (radium element) from two hundred eighty to eleven thousand two hundred milligram hours radiation (radium element) during the course of treatment. This would require a much larger amount of radium and without it no one should expect satisfactory results except in small superficial rodent ulcers or epithelioma, seventeen of which Dr. Beck reports as cured with ten to twenty milligrams. If the Doctor would give more thought to radium-therapy and use adequate quantities of radium, I believe he would be able to cure more "deeper seated malignant disease, particularly carcinoma," as well as rodent ulcer and epithelioma. This "damning with faint praise" by those who presumably have worked with radium but who really have attempted too much with the radium available or have used a faulty technique, is most unfair to those using radium. Such ill-advised work will retard the development of radium-therapy far more than the criticism of the frankly sceptical men who have had neither a personal experience with radium nor an opportunity to observe the results that can be attained with it. For this reason in citing case reports it is necessary to give exact data with regard to the amount of radium element used, the time of application, etc., since a failure to obtain a good result may often be due to inadequate dose or poor technique.

The doses in the above cases were repeated from three to six weeks apart and the milligram hours radiation (radium element) per case varied from two hundred forty in rodent ulcers to fifteen thousand milligram hours' radiation (radium element) in the most aggravated cases. The periods of treatment were from one to eight months. Although it is too early to state final resultst the patients so far as known are in excellent condition at the present time.

I have herein cited nineteen of the better cases, only one of which was operable; two died, one is still doubtful, and sixteen are clinically cured.

179 Lake Avenue.

---

**Pediculicide.** Through the mistake of an assistant in substituting anisol for anise oil, Prof. Sigmund Fraankel has been able to announce to the Vienna Medical Society that the former is an efficient destroyer of lice, which have assumed an important place in military hygiene through carrying typhus germs.

---

**Crotalin in Epilepsy.** W. J. Chening of The Plains, Va., May 1915, Charlotte Med. Jour., reports very favorable results in a severe case. In view of the generally unfavorable reports, this is worth noting.

